# Nilotinib-Induced Dystonia and Cognitive Deficits in a Neurologically Normal Patient with Chronic Myeloid Leukemia

**DOI:** 10.1155/2019/3679319

**Published:** 2019-11-12

**Authors:** Justine Chan, Paarth Shah, Guillermo Moguel-Cobos

**Affiliations:** Department of Neurology, Barrow Neurological Institute, St. Joseph's Hospital and Medical Center, Phoenix, AZ, USA

## Abstract

Nilotinib is a tyrosine kinase inhibitor used to treat patients with chronic myeloid leukemia (CML). This agent is also being studied in neurodegenerative disorders including Parkinson disease. Studies have shown that nilotinib may decrease the accumulation of parkin substrates and decrease the loss of dopaminergic cells. The use of nilotinib in neurologic disorders is relatively new, and little information about this use has been published. We report on a patient receiving nilotinib for CML. The patient had no previous neurologic deficits, and developed intermittent dystonic posturing of the left upper extremity and cognitive impairment after she began nilotinib treatment. The mechanisms behind this adverse effect are not clear; however, her symptoms began after nilotinib was introduced, decreased with dose reduction, stopped with its cessation, and re-emerged when the medication was restarted. To our knowledge, this is the first reported patient with neurologic symptoms secondary to nilotinib use.

## 1. Introduction

Nilotinib is a tyrosine kinase inhibitor used to treat patients with chronic myeloid leukemia (CML) by deactivating the Philadelphia chromosome, which contains the fusion gene *BCR-ABL*. The *BCR-ABL* gene is also present in patients with Parkinson disease (PD) [1]. Over-activation of tyrosine kinase may indicate increased oxidative stress, which may play a role in the loss of dopaminergic neurons contributing to the pathogenesis of PD. Nilotinib has been studied in PD patients because it may decrease the accumulation of parkin substrates and decrease the loss of dopaminergic cells. We report on a patient receiving nilotinib for CML who developed intermittent dystonic posturing of the left upper extremity and cognitive impairment. To our knowledge, this is the first reported patient with neurologic symptoms believed to be secondary to nilotinib use.

## 2. Case Presentation

A 38-year-old woman, with a history of CML, presented for evaluation of abnormal movements that began several months prior. Clinical examination showed speech difficulty, cognitive deficits, intermittent left upper extremity dystonic posturing (elevation, adduction and flexion of arm and hand, fist posturing) and head tilting to the left. The remainder of her neurologic examination was normal.

She had been diagnosed with CML 3 years previously and was initially treated with imatinib, but she was later transitioned to nilotinib 300 mg twice a day because of lack of response to imatinib. Within a few weeks of starting nilotinib, her neurologic symptoms appeared. Months later, nilotinib was discontinued, and her neurologic symptoms resolved. She remained free from neurologic symptoms until nilotinib was restarted at a lower dose (150 mg twice daily), then the neurologic symptoms returned again after a few weeks. A further reduction of nilotinib dose to 150 mg once daily resulted in significant amelioration of her symptoms, and she remains this way clinically.

Results from magnetic resonance imaging of the patient's brain with and without contrast, electroencephalography, a serum paraneoplastic panel, and cerebrospinal fluid analysis while she was on the higher dose of nilotinib (ie, 300 mg twice daily) were normal. Neuropsychological testing, performed while the patient was on the higher dose of nilotinib, showed mild cognitive impairment with frontal lobe and executive function deficit. Results of FDG positron emission tomography (PET) when she was on the lower dose (150 mg twice daily) were also normal. Also noted were impairments in her semantic verbal fluency and verbal processing speed, and she exhibited intermittent dysarthric speech.

## 3. Discussion

This patient with CML had no previous neurologic deficits but developed neurologic symptoms after she began treatment with nilotinib. The mechanisms behind this adverse effect are not clear, but her symptoms began after medication was introduced, decreased with dose reduction, stopped with its cessation, and re-emerged when the medication was restarted.

Nilotinib is a selective breakpoint cluster region-Abelson (BCR-ABL) tyrosine kinase inhibitor developed for CML patients who are resistant to treatment with imatinib [1, 2]. Nilotinib is more potent and selective than imatinib, has improved brain penetration, and is considered one of the most effective drugs for treatment of CML today [2]. The Abelson nonreceptor tyrosine kinase (c-Abl) is vital for physiologic cellular processes, including regulation of the cell cycle and DNA repair [1, 3, 4].

Parkinson disease is a neurodegenerative disorder characterized by loss of dopaminergic neurons in the substantia nigra neurons and accumulation of alpha synuclein protein. PD models have suggested that overactivation of c-Abl tyrosine kinase phosphorylation leads to loss of the ligase activity of parkin and thus leads to mitochondrial dysfunction and the accumulation of toxic substrates and synuclein [3–5]. Mouse models of PD have shown that nilotinib clears both brain and blood alpha-synuclein, whose gene encodes for alpha-synuclein, and it reverses the loss of tyrosine hydroxylase-positive neurons [6]. c-ABL is also activated in 1-methyl-4-phenyl-1,2,3,6-tetrahydropyridine (MPTP) mice [3]. Evidence from MPTP models of PD has suggested that nilotinib reduces c-ABL activation and further prevents dopamine neuron loss by reducing the parkin substrate, PARIS, independent of reduction of tyrosine phosphorylation of parkin [3]. These animal models suggest a potential neuroprotective effect of nilotinib; however, this concept still needs to be evaluated in human studies. Nilotinib's importance in PD is believed to be due to its ability to penetrate the blood-brain barrier and degrade misfolded alpha-synuclein via autophagy, as well as to inhibit BCR-ABL and reduce oxidative stress, thus protecting dopamine neurons and leading to improvement in motor and cognitive outcomes [3, 5]. However, this theory has not been tested in those unaffected by PD, and our reported adverse effect of dystonia and cognitive deficits has not been previously associated with this tyrosine kinase inhibitor.

Pagan et al. [7] conducted a small study of 12 patients with PD dementia and dementia with Lewy bodies and found that nilotinib at small doses was safe and tolerated, with good cerebrospinal fluid penetration, and with some possible benefit in motor and cognitive outcomes. This study prompted a larger study by Pagan that is currently recruiting toward a goal of 60 patients to evaluate the safety, tolerability, and pharmacokinetics of nilotinib in patients with PD; this study is projected to end in 2023. The NILO-PD study is a Phase 2a randomized, double-blind, placebo-controlled cohort study looking at nilotinib in 135 patients at low doses in those with PD and its safety and tolerability. Secondary outcomes will assess cognitive and motor effects [8]. The nilotinib dosage used in these trials (150–300 mg/day) is much lower than the FDA-approved dosage for the treatment of CML (600–800 mg/day). Severe adverse effects have been reported for this drug when used for CML and have primarily consisted of cardiac symptoms [7].

While our patient had no symptoms of parkinsonism, it is possible that parkin substrates had accumulated and could have led to functional modifications of her basal ganglia, leading to dystonia. However, it is also likely that she experienced a symptomatic effect of nilotinib due to altered central nervous system dopamine levels as suggested by Pagan et al. [7]. At 150 mg/day and 300 mg/day dose, Pagan and colleagues found increased levels of cerebrospinal fluid homovanillic acid (a dopamine metabolite), suggesting a direct alteration of dopamine metabolism [7].

Other rare neurologic adverse effects that have been reported with BCR-ABL inhibitors include rapidly progressive vasculopathy as well as dasatinib-associated reversible demyelinating peripheral polyneuropathy [9, 10]. Nilotinib has been associated with progressive peripheral arterial disease and ischemic heart disease, and a few cases have been reported of intracranial vasculopathy, such as that described by Chen et al. [9], with an atypical case of rapid intravascular non-atherosclerotic arterial stenosis. That case report reviews varying etiologies that could explain nilotinib's prothrombotic state, including expression of cell adhesion molecules that promote a pro-atherogenic phenotype [9]. This is certainly a possible explanation of symptoms in our case. A 4-vessel cerebral angiogram should have been performed, but was not in our patient. As noted, the brain FDG PET revealed normal glucose metabolism globally and, although this procedure can be used as a test for blood flow and metabolism, the lack of a cerebral angiogram is certainly a limitation to our case report. Given that our patient had no sensory deficits, an electromyography and nerve conduction study were not conducted. It is important to be mindful that medications that cause a peripheral neuropathy are usually distal, symmetric, and axonal, and cause a sensory neuropathy. However, there are rare cases of drug-induced peripheral neuropathy, such as that described by Ishida et al. [11].

Regarding our patient's cognitive changes, we theorize that it is related to nilotinib, on the basis of the timing of her symptoms in correlation with medication and the dose-dependent correlation of her symptoms. It is possible that alteration of dopamine levels may play a role in changes in cognition or clearance of intraneuronal phosphorylated tau and alpha synuclein by nilotinib itself [12]. As mentioned above, on one hand, a cerebral blood flow limitation is also another possibility for our patient's symptoms. On the other hand, the small clinical study by Pagan also suggests cognitive improvement with low doses of this tyrosine kinase inhibitor.

Another limitation of this report is that nilotinib levels in the cerebrospinal fluid were not directly measured to provide a correlation with the patient's symptoms. In addition, no measurements of cerebrospinal fluid disease-related biomarkers such as alpha synuclein were taken.

## 4. Conclusions

Nilotinib is currently used in CML patients to deactivate BCR-ABL kinase. In PD, nilotinib is thought to act by reducing harmful parkin substrates, thus preventing dopaminergic neuron cell loss. We report the first case known to demonstrate nilotinib causing intermittent dystonic posturing, dysarthric speech, and cognitive impairment in a neurologically normal patient. The pathophysiology underlying this adverse effect is most likely associated with disruption of the alternate protein kinases that are responsible for the proliferation and transduction of normal signals through the basal ganglia or by a direct effect on dopamine levels. As nilotinib is further studied for its usefulness in PD, we believe that its adverse effect profile and dosage should be carefully considered in a randomized, double-blind trial that includes a placebo group. This is important, as c-ABL inhibitors such as nilotinib may have a role in treating other neurodegenerative diseases, such as Alzheimer disease, and human tauopathies given c-ALB over-activation has been implicated in the pathogenesis of these neurodegenerative diseases [13].

## Abbreviations

CML: Chronic myeloid leukemia
MPTP: 1-methyl-4-phenyl-1,2,3,6-tetrahydropyridine
PD: Parkinson disease.
